# PAA Modified Upconversion Nanoparticles for Highly Selective and Sensitive Detection of Cu^2+^ Ions

**DOI:** 10.3389/fchem.2020.619764

**Published:** 2021-01-08

**Authors:** Shaoshan Su, Zhurong Mo, Guizhen Tan, Hongli Wen, Xiang Chen, Deshmukh A. Hakeem

**Affiliations:** Key Laboratory of Clean Chemistry Technology of Guangdong Regular Higher Education Institutions, School of Chemical Engineering and Light Industry, Guangdong University of Technology, Guangzhou, China

**Keywords:** energy transfer, copper ions, fluorescent probe, poly(acrylic acid) (PAA), upconversion nanoparticles (UCNPs)

## Abstract

Detection of the Cu^2+^ ions is crucial because of its environmental and biological implications. The fluorescent-based organic sensors are not suitable for Cu^2+^ detection due to their short penetration depth caused by the UV/visible excitation source. Therefore, we have demonstrated a highly sensitive and selective near-infrared (NIR) excitable poly(acrylic acid) (PAA) coated upconversion nanoparticles (UCNPs) based sensor for Cu^2+^ detection. We construct the PAA modified Na(Yb, Nd)F_4_@Na(Yb, Gd)F_4_:Tm@NaGdF_4_ core-shell-shell structured UCNPs based sensor via a co-precipitation route. The upconversion emission intensity of the PAA-UCNPs decreases linearly with the increase in the Cu^2+^ concentration from 0.125 to 3.125 μM due to the copper carboxylate complex formation between Cu^2+^ and PAA-UCNPs. The calculated detection limit of the PAA-UCNPs based sensor is 0.1 μM. The PAA-UCNPs based sensor is very sensitive and selective toward detecting the Cu^2+^ ions, even when the Cu^2+^ co-exist with other metal ions. The EDTA addition has significantly reversed the upconversion emission quenching by forming the EDTA-Cu^2+^ complex based on their greater affinity toward the Cu^2+^. Therefore, the PAA-UCNPs based sensor can be a promising candidate for Cu^2+^ detection because of their higher sensitivity and selectivity under 980 nm NIR excitation.

## Introduction

To detect the trace elements, both essential (Cr, Co, Cu, Fe, Li, Mg, Mn, Ni, Se, and Zn) and non-essentials (P and S), developing highly selective and sensitive sensors is a crucial, useful and challenging task in the fields of medical, environment, and biology (Helal et al., 2011). These elements are essential for numerous biological processes in living organisms; however, their excessive accumulation in the human body may lead to detrimental effects. A trace of these elements plays a crucial role by acting as a cofactor of enzymes in biological activities, e.g., the Mn ions regulate the physiological processes, including electron transport, oxygen transportation, protein modification, and neurotransmitter synthesis (Chen P. et al., 2016). These elements can accumulate in the brain, which affects the nervous system and catalyzes cytotoxic reactions at high concentrations. These ions anomaly could result in neurological disorders, heart attack, breathing problems, pseudotumor cerebri, and cranial nerve palsy related to Fe ions deficiency (Chen P. et al., 2016).

Among these elements, the copper (Cu^2+^), being the third most essential and abundant micronutrient, exists in very low concentration and participates in various organism's physiological processes (Becker et al., 2005; Sikdar et al., 2018). Mostly, Cu^2+^ resides in the brain, particularly in basal ganglia, hippocampus, cerebellum, and numerous synaptic membranes (Vishal Desai, 2008). Several enzymes require Cu^2+^ for their functioning, such as tyrosinase, peptidyl glycine amidating monooxygenase, copper/zinc superoxide dismutase, ceruloplasmin, hephaestin, dopamine-hydroxylase, and cytochrome c oxidase (Vishal Desai, 2008). The anomaly in Cu^2+^ concentration may lead to multiple disorders (Jiang and Meng, 2013). The deficiency of Cu^2+^ could cause anemia, coronary heart diseases, and bone abnormalities (Fu et al., 2019). Simultaneously, the excess Cu^2+^ can initiate oxidative stress, leading to neurodegenerative diseases such as Menkes syndrome, Indian childhood cirrhosis, and Wilson's, Alzheimer's, Parkinson's, and prion diseases (Vishal Desai, 2008; Chen P. et al., 2016; Sikdar et al., 2018). Excessive Cu^2+^ consumption could cause kidney/liver damage, amyotrophic lateral sclerosis, and vomiting (Sikdar et al., 2018). On the other hand, Cu^2+^ generated as industrial waste is responsible for the marine environment pollution, thereby contaminating marine organisms, ultimately affecting human health through poisoning if the polluted marine organisms are consumed (Fu et al., 2019). Therefore, it is necessary to develop a highly sensitive and selective fluorescent probe for Cu^2+^ detection and quantification in environmental and biological samples.

The presence of trace amount of Cu^2+^ can be quantitatively estimated via traditional analytical techniques such as atomic absorption spectrometry (AAS) (Tokay and Bagdat, 2015), atomic emission spectrometry (AES) (Atanassova and Russeva, 1998), inductively coupled plasma mass spectrometry (ICP-MS) (Becker et al., 2005), colorimetry (Liu et al., 2018), solid-phase extraction (SPE) (Liu et al., 2020), voltammetry (Liu et al., 2020), and fluorescence spectrometry (Zhang et al., 2014). Colorimetry is mostly applied in paper-based analytical devices (PADs) for low-cost, fast, and simple analysis (Cate et al., 2015; Ostad et al., 2017). However, the detection capability or sensitivity of the colorimetric technique is not good (Liu et al., 2018). In voltammetry, the generally used toxic mercury and mercury coated electrodes (Sonthalia et al., 2004) are interchanged with non-toxic platinum (Bu et al., 2015), and screen printed carbon (Chaiyo et al., 2016) electrodes, which produce high background current during the detection, thus become un-appropriate for accurate detection (Liu et al., 2020). The above techniques relied on expensive equipment, complex testing procedures, and poor sensitivity, limiting its bio-application (Xu et al., 2016).

Fluorescence spectroscopy uses fluorescent probes as a detection tool to develop highly sensitive, selective, low cost, and simple methods for detecting Cu^2+^. Up to now, most of the reported fluorescent probes are organic such as rhodamine-based derivatives (Tang et al., 2011), BODIPY-based derivatives (Loudet and Burgess, 2007), coumarin-based derivatives (Sheng et al., 2008), and naphthalimide-based fluorogenic probe (Xu et al., 2005; Zhang et al., 2014; Fu et al., 2019). These organic fluorescent probes absorb high energy UV/blue light, which resulted in low detectability due to the background fluorescence interference and shallow penetration depth (Jiang and Meng, 2013; Ostad et al., 2017). The nominal concentration of the Cu^2+^ in blood and drinking water allowed by the U.S. Environmental Protection Agency (EPA) is around 100–150 μg/dL (15.7–23.6 μM) and 1.3 ppm (~20 μM), respectively (Helal et al., 2011; Jiang and Meng, 2013; Sikdar et al., 2018). Similarly, the World Health Organization (WHO) has announced that the oral intake of Cu^2+^ could not exceed more than a few milligrams (2 or 3 mg/day) in adults (Fu et al., 2019). Recently, the fluorescence whitening agent was used in paper-based sensors to achieve 160-fold improved Cu^2+^ detection with 69 nM detection limit (Liu et al., 2018). At the same time, the separation of the metal ions and the complication entities through a reaction boundary on a paper-based sensor achieve a detection limit of 10 mM. The Cu^2+^ ions with a detection limit of 12.5 μM can be detected via a reaction between metal ions and color developing agents. Another sensor for the Cu^2+^ detection was developed based on fluorophore (dansyl moiety) and a multidentate ligand. The reaction between the probe with Cu^2+^ produces a stable Cu^2+^ complex that quenches the fluorophore's emission. This method detects the Cu^2+^ up to 2 ppb (Zhang et al., 2014).

Upconversion nanoparticles (UCNPs), which convert the near Infrared (NIR) light (generally 808 or 980 nm) into visible light, have been become a promising candidate for the bio detection application. Compared with the dyes and quantum dots, the UCNPs exhibit several benefits: sizeable anti-Stokes shift, low toxicity, high chemical/photostability, lower photodamage, and deeper light-penetration depth (Zhang S. et al., 2018), establishing themselves as a promising candidate for bio detections application. Lanthanide (Ln^3+^) doped UCNPs exhibit several intra 4f−4f transitions producing strong tunable emissions of different colors with longer lifetimes by changing the size and morphology of the UCNPs. Also, the UCNPs can produce tunable multi-color emissions via controlling the dopants concentrations. Low energy NIR light excitation provides a high sensitivity detection system with negligible auto-fluorescence and light scattering. In this regards, different research groups have developed UCNPs based sensors for the detection of organic and inorganic entities such as toluene/phenol (Ma et al., 2014), organophosphorus (Wang et al., 2016), Zinc (Han et al., 2010), Mercury (Li et al., 2014; Wu et al., 2016), Cyanide (Liu et al., 2011), and other entity (Huang et al., 2017).

Most of the UCNPs are hydrophobic because they are generally prepared by the thermal decomposition of the precursors (metal trifluoroacetate) in organic solvents (oleic acid (OA)/oleylamine/1-octadecene). Besides, the bare UCNPs induces the quenching effect due to the surface defects and vibrational deactivation from solvents (Wang et al., 2009; Zhu et al., 2013). Therefore, hydrophilic UCNPs with minimum quenching effect is desirable for efficient bio-detection. The surface modification overcomes the above limitations through well-known core-shell strategy and coating reactive functional moieties layer on UCNPs, which are excellent methods to minimize the quenching effect thereby significantly improving the luminescence intensity of UCNPs via the reduction in surface defects and non-radiative loss (Chen et al., 2012). Various groups have reported the core-shell UCNPs, such as NaYF_4_:Yb,Er@NaGdF_4_ and NaYbF_4_:Er/Tm@NaGdF_4_ (Su et al., 2012; Zhang et al., 2012; Jin et al., 2013). Recently, Chen et al. (Chen X. et al., 2016) investigated the hexagonal phase NaYF_4_@NaYbF_4_:Tm@NaYF_4_ core-shell structure with Yb^3+^ ions enclosed in the inner shell layer. The emission increased up to five-photon upconversion of Tm^3+^ due to the confined energy migration in NaYF_4_@NaYbF_4_:Tm@NaYF_4_ core-shell structure. Yb^3+^ sensitizer highly absorbs NIR light (~980 nm) and Yb^3+^ concentration can reach up to ~100% in hexagonal NaYF_4_:Yb^3+^/Tm^3+^@NaYF_4_ core/shell nanocrystals after overcoming concentration quenching of Yb^3+^ (Ma et al., 2017; Pliss et al., 2017). Homann et al. (2018) investigated the quantum yields of the NaYF_4_:Yb/Er@NaYF_4_ nanoparticles by changing the NaYF_4_ shell thickness. The increase in the thickness of NaYF_4_ shell from 15 to 45 nm, increases the quantum yield from 3.4 to 9%. A similar study was performed by Würth et al. (2018) on hexagonal NaGdF_4_:0.2Yb/0.02Er UCNPs upon altering the NaYF_4_ shell thickness. Recently, Zhou et al. (2020) developed a core-shell-shell triple-layered NaYF_4_:Er@NaYbF_4_@NaYF_4_ structure. Compared to NaYF_4_:Yb,Er@NaYF_4_, the upconversion emission intensity of the α-NaYF_4_:10%Er@NaYbF_4_@NaYF_4_, where the Er^3+^ and Yb^3+^ were doped separately in the core and intermediate layer, was significantly increased, ~100 times.

In UCNPs based sensors, the UCNPs serve as the energy donors, while, the target chromophores act as energy acceptors (Huang et al., 2017). Until now, the UCNPs are employed as energy donors and the family of rhodamine B (RhB) as energy acceptor in the Förster resonance energy transfer (FRET) based Cu^2+^ detection. Jiang and Meng (2013) made a sensor by combining β-NaYF_4_:Yb^3+^/Er^3+^ (UCNPs) and 2-amino-3′,6′-bis(ethylamino)-2′,7′-dimethyl-3′,9a′-dihydrospiro[isoindoline-1,9′-xanthen]-3-one (a rhodamine derivative) based on energy transfer process between UCNPs and rhodamine derivative. The rhodamine derivative was excited by the UCNPs, and detected Cu^2+^ in the 2–14 μM range with good selectivity and high chemo stability. The core-shell technology also helps to improve the sensitivity of Cu^2+^ detection. Zhang Y. et al. (2018), reported Cu^2+^ detection using core-shell NaYF_4_@NaYF_4_:Er^3+^/Yb^3+^@NaYF_4_ UCNPs and RhB hydrazide as donor and acceptor, respectively. NaYF_4_@NaYF_4_:Er^3+^/Yb^3+^@NaYF_4_ has higher emission intensity than the bare NaYF_4_:Er^3+^/Yb^3+^ UCNPs, which has enhanced the selectivity and specificity of the Cu^2+^ detection. Li et al. (2012) used RhB hydrazide doped porous SiO_2_ shell to protect UCNPs. The addition of Cu^2+^ ions in UCNPs dispersion showed the color change and improved emission of RhB hydrazide–Cu^2+^ complex under 980 nm excitation, also, the green emission of the UCNPs decreased. However, these sensors exhibit low emission when dispersed in water, using RhB-hydrazide dye, silica shell and most importantly, the sensors involved are not reusable.

**Scheme 1:**
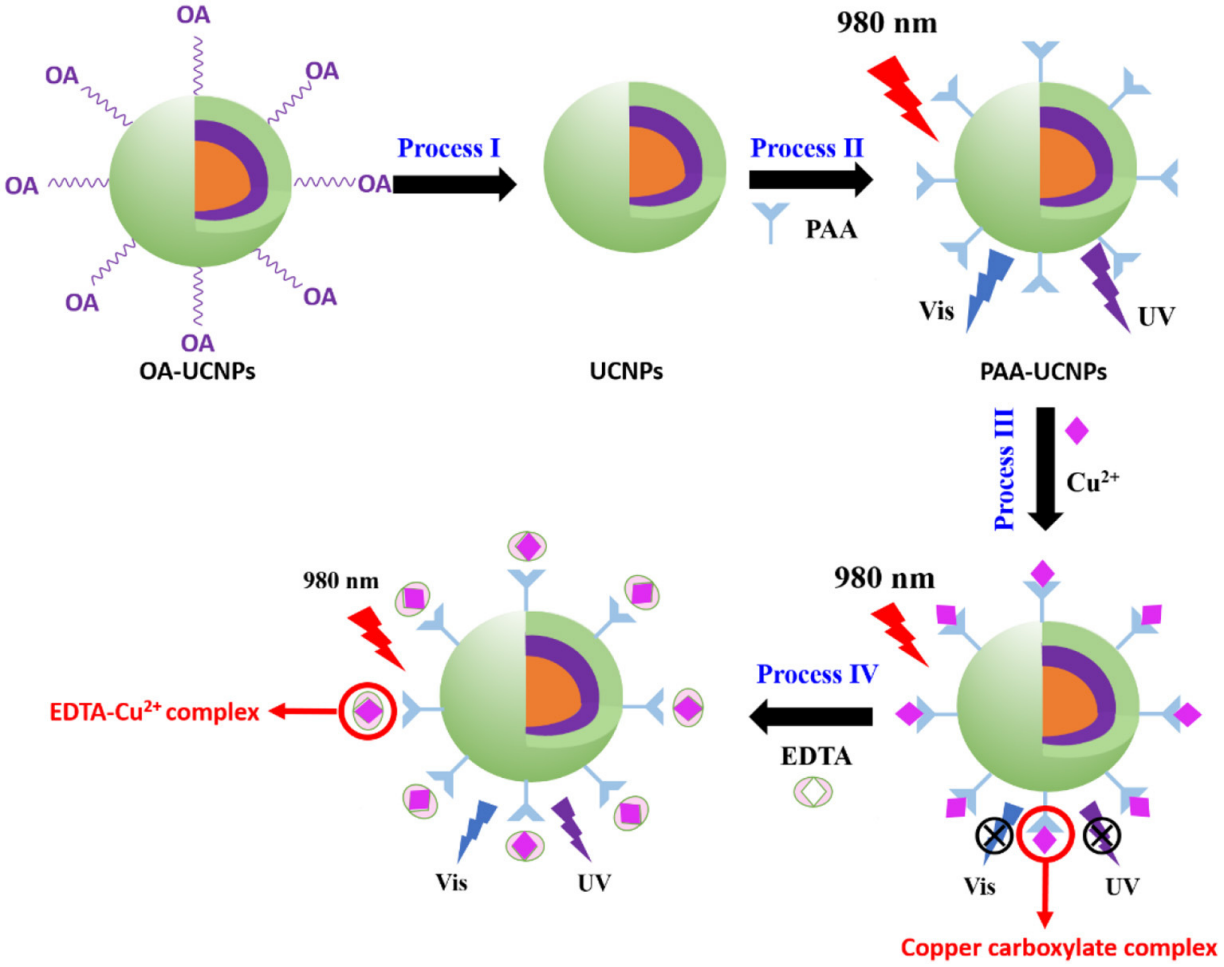
Principle of Cu^2+^ detection based on energy transfer mechanism using core-shell-shell upconversion nanoparticles (UCNPs).

In this study, we construct a new upconversion sensor for Cu^2+^ detection using water-dispersible poly(acrylic acid) (PAA) functionalized Na(Yb,Nd)F_4_@Na(Yb,Gd)F_4_:Tm@NaGdF_4_ core-shell-shell structured UCNPs, where the UCNPs work as the energy donor and Cu^2+^ ions as the acceptor. The reversible process of Cu^2+^ detection comprising the fluorescence quenching and recovery upon adding Cu^2+^ ions and EDTA, respectively, is schematically illustrated in Scheme 1. Efficient Na(Yb,Nd)F_4_@Na(Yb,Gd)F_4_:Tm@NaGdF_4_ UCNPs, composed of NaYb_0.5_Nd_0.5_F_4_ core, NaYb_0.5_Gd_0.49_Tm_0.01_F_4_ first shell and NaGdF_4_ second shell were prepared by using co-precipitation method. At first, the OA ligand, attached during the preparation, was removed, followed by functionalization with PAA to form PAA-UCNPs for realizing efficient Cu^2+^ detection (Process I and II). With Cu^2+^ (Process III), the carboxylate group of PAA-UCNPs can bind selectively to the Cu^2+^ ions owing to the electrostatic interaction between Cu^2+^ and carboxylate anion of PAA, forming copper carboxylate complex to quench the UCNPs fluorescence. Upon 980 nm NIR light excitation, in the presence of Cu^2+^, the emission intensity of the UCNPs was quenched linearly as the Cu^2+^ concentration increased from 0.125 to 3.125 μM with the detection limit of 0.1 μM. The upconversion emission intensity of the UCNPs was recovered up to 90% after further addition of EDTA (Process IV). Besides, the current UCNPs based nano-platform showed high sensitivity and selectivity of Cu^2+^ ions over the range of other metal ions (Al^3+^, Ba^2+^, Ca^2+^, Co^2+^, K^+^, Li^+^, Mg^2+^, Mn^2+^, Na^+^, Ni^2+^, and Zn^2+^).

## Experimental Sections

### Material and Reagents

Following chemicals with appropriate purities were used as received without further purification. Nd(CH_3_CO_2_)_3_ · *x*H_2_O, 99.9%; Yb(CH_3_CO_2_)_3_ · 4H_2_O, 99.9%; Gd(CH_3_CO_2_)_3_ · *x*H_2_O, 99.9%, Tm(CH_3_CO_2_)_3_ · *x*H_2_O, 99.9%; NaOH, >98%; NH_4_F, >98%; 1-octadecene, 90%; oleic acid, 90%; poly(acrylic acid) (PAA), MW ≈ 1800; HCl, >98%, were purchased from Sigma-Aldrich. C_10_H_14_N_2_O_8_Na_2_ · 2H_2_O (EDTA) (>98%) were purchased from Sangon Biotech, China. Ethanol (99.5%), cyclohexane (99.5%), diethylene glycol (DEG) (>99%) were purchased from Macklin, China. The solutions of metal cations were performed from their corresponding salts of analytic grade or molecular biology grade, such as LiCl, NaCl, KCl, MgCl_2_·6H_2_O, CaCl_2_·4H_2_O, AlCl_3_·6H_2_O, BaCl_2_, ZnCl_2_, MnCl_2_·4H_2_O, CoCl_2_·6H_2_O, NiCl_2_·6H_2_O, and CuCl_2_·3H_2_O. The water used in this work is ultrapure water (18.2 MΩ cm) deionized by the Milli-Q system.

### Sample Synthesis

#### Preparation of Core UCNPs

The core UCNPs were synthesized by using a modified co-precipitation method, as reported elsewhere (Wang et al., 2011; Wen et al., 2013). In a typical procedure to the synthesis of NaYb_0.5_Nd_0.5_F_4_ core nanoparticles, 2-mL water solution of Nd(CH_3_CO_2_)_3_ (0.2 M) and Yb(CH_3_CO_2_)_3_ (0.2 M) was mixed with 4 mL of oleic acid and 6 mL of 1-octadecene solution in 50-mL flask. The mixture was heated at 120°C for 30 min, then heated at 170°C for 40 min to form the lanthanide-oleate complexes and then cooled down to room temperature. Thereafter, mixed methanol solution of NH_4_F (1.5 mmol) and NaOH (1 mmol) was added to the above mixture and heated at 45°C for 30 min. After methanol evaporation at 110°C, the solution temperature was raised to 295°C and maintained for 1.5 h under argon. Post 295°C heating, the solution was cooled to room temperature and then the resulting nanoparticles were washed with ethanol and cyclohexane for several times and re-dispersed in 6.5 mL of cyclohexane.

#### Preparation of Core-Shell UCNPs

In a typical procedure to the shell growth of NaYb_0.5_Gd_0.49_Tm_0.01_F_4_ layer, the shell precursor were synthesized by adding 2 mL water solution of Ln(CH_3_CO_2_)_3_ (0.2 M, Ln = Yb,Gd,Tm), 4 mL oleic acid and 6 mL 1-octadecene to a 50 mL flask, and heated at 120°C for 30 min, then heated at 170°C for 40 min to form the lanthanide-oleate complexes, and then cooled down to room temperature. The preformed core nanoparticles dispersed in 3 mL of cyclohexane with methanol solution of NH_4_F (1.5 mmol) and NaOH (1 mmol) were added and heated to 45°C for 30 min. After methanol evaporation, the reactant was heated at 295°C for 1.5 h under argon atmosphere followed by cooling to room temperature. The resulting nanoparticles were washed several times with ethanol and cyclohexane, and re-dispersed in 4 mL of cyclohexane. The procedures were repeated for the preparation of core-shell-shell nanoparticles.

#### Preparation of Uncapped UCNPs (Kong et al., 2017)

The oleate capped UCNPs (denoted as OA-UCNPs) dispersed in 2 mL of cyclohexane were precipitated by ethanol and dispersed again in 2 mL of HCl solution (0.1 M). The mixture was then sonicated at 35°C for 1 h to remove the OA ligands. After the reaction, the uncapped UCNPs were collected via centrifugation for 0.5 h (at 15,000 rpm), followed by washing twice with deionized water and re-dispersed in deionized water (2 mL, 0.25 M).

#### Preparation of PAA Capped UCNPs (Kong et al., 2017)

Typically, 30 mg of poly acrylic acid (PAA) was added to the deionized water (9 mL) with NaOH (0.1 M in deionized water) to adjust the pH at 8 under robust stirring at room temperature, followed by the addition of uncapped UCNPs (1 mL) dropwise, and the mixture was stirred for another 2 h. This solution was then mixed with DEG (10 mL) and heated at 105°C for 1 h under stirring for water evaporation. The resultant solution was then heated at 160°C for 2 h in a 20 mL Teflon-lined autoclave. Next, the PAA capped UCNPs (denoted as PAA-UCNPs) were collected through centrifugation at 15,000 rpm for 0.5 h. PAA-UCNPs were washed several times with deionized water and ethanol, re-dispersed in deionized water (1 mL, 0.25 M), and stored at 4°C in fridge.

### Characterization

To investigate the morphology/size of UCNPs, the samples were characterized by a HT7700 transmission electron microscope (TEM) with the working voltage of 100 KV (Hitachi, Japan). The crystal phase of UCNPs was identified by DY735 X-ray diffractometer (XRD) (Malvern Panalytical company, UK) with 2θ range from 10 to 80° at a scanning rate of 5° per minute, with Cu Kα irradiation (λ = 1.5406 Å). Fourier transform infrared (FT-IR) spectra of UCNPs using KBr pellet technique were recorded between 400 and 4,000 cm^−1^ at a resolution of 1 cm^−1^ using a Nicolet 6700 FT-IR Spectrometer (Thermo-Fisher Scientific, USA). Ultraviolet-visible (UV–Vis) spectra were measured on SHIMADZU UV2700 spectrophotometer. Upconversion luminescence spectra were recorded by FluoroMax-4 fluorescence spectrometer (HORIBA Jobin Yvon, USA) at the same condition, except using an external 980 nm CW laser at 3W or 808 nm laser at 2 W (Fiber coupled diode laser, BWT Beijing Ltd.). The photoluminescence intensity was obtained by integration the upconversion emission spectra in wavelengths range of 330–850 nm. Thermogravimetric analysis (TGA) was performed using Netzsch DTA-TG STA449F5 instrument under N_2_ atmosphere at a heating rate of 10° per minute.

### Sample Preparation for Metal Ion Sensing

The metal ion stock solutions with a concentration of 5 × 10^−3^ M were prepared in deionized water using the corresponding metal salts and diluted in deionized water at concentrations of 5 × 10^−4^ M and 5 × 10^−6^ M, respectively. The PAA-UCNPs dispersion with concentrations of 0.25 and 0.025 M were prepared in deionized water. The PAA dispersion with a concentration of 5 × 10^−3^ M was prepared in deionized water. The EDTA stock solution with a concentration of 5 × 10^−3^ M was prepared in deionized water.

In the UV-Vis experiment of Figure 2A, 2 mL PAA-UCNPs dispersion (0.025 M) was diluted to 4 mL in deionized water; 8 μL PAA-UCNPs dispersion (0.025 M) was mixed with 160 μL Cu^2+^ solution (5 × 10^−4^ M) and diluted to 4 mL in deionized water. In the UV-Vis experiment of Figure 2B, the PAA (5 mM), Cu^2+^ (5 μM) and PAA (40 μM) chelating with Cu^2+^ (40 μM) were prepared in deionized water by diluting corresponding bulk solutions. The mixture was filled in a quartz cell with an optical path of 1.0 cm to achieve an absorption spectrum. For the upconversion emission experiment with 980 nm laser excitation, 40 μL PAA-UCNPs dispersion (0.025 M) was added to a 4 mL bottle, then the Cu^2+^ solution (5 × 10^−6^ M) was added by means of a micro-pipette and diluted to 4 mL. The mixture was filled in a quartz cell with an optical path of 1.0 cm for testing. For the upconversion emission experiment with 808 nm laser excitation, 100 μL PAA-UCNPs dispersion (0.25 M) was added to a 3 mL bottle, then the Cu^2+^ solution (5 × 10^−6^ M) was added by means of a micro-pipette and diluted to 3 mL. The mixture was filled in a quartz cell with an optical path of 1.0 cm for testing. For upconversion emission spectra of the selective experiment with 980 nm laser excitation, 40 μL PAA-UCNPs dispersion (0.025 M) was added to a 4 mL bottle, then the metal ion solution (5 × 10^−6^ M or 5 × 10^−4^ M, respectively), was added by means of a micro-pipette and diluted to 4 mL. The mixture was filled in a quartz cell with an optical path of 1.0 cm for testing. For upconversion emission spectra of the reversibility experiment, EDTA was added dropwise to the solution of PAA-UCNPs whose fluorescence has been quenched by copper ions. After mixed well, filled it into a quartz cell with a 1.0 cm optical path for testing. After recovery experiment through addition of EDTA, the PAA-UCNPs were collected via centrifugation, followed by washing several times with deionized water and ethanol, re-dispersed in deionized water, and stored at 4°C in fridge for another Cu^2+^ sensing test.

### Calculation of Limit of Detection

The limit of detection, LOD, of UCNPs chelating with Cu^2+^ was determined using the following equation (Peng et al., 2009; Guo et al., 2017):

(1) $$\begin{array}{l} \text{LOD}=3\times \delta /S \end{array}$$

(2) $$\begin{array}{l} \delta =\sqrt{\frac{\Sigma \text{(}{\text{x}}_{i}\text{- x)}{\;}^{\text{2}}\;}{N}} \end{array}$$

where δ is the standard deviation of the blank solution, *S* is the slope of the calibration curve, *x*_*i*_ is the average value from the calculation, *x* is the measured data point, and *N* is the number of readings employed for obtaining average.

## Results and Discussion

### Structural Characterizations and Morphology

**Figure 1:**
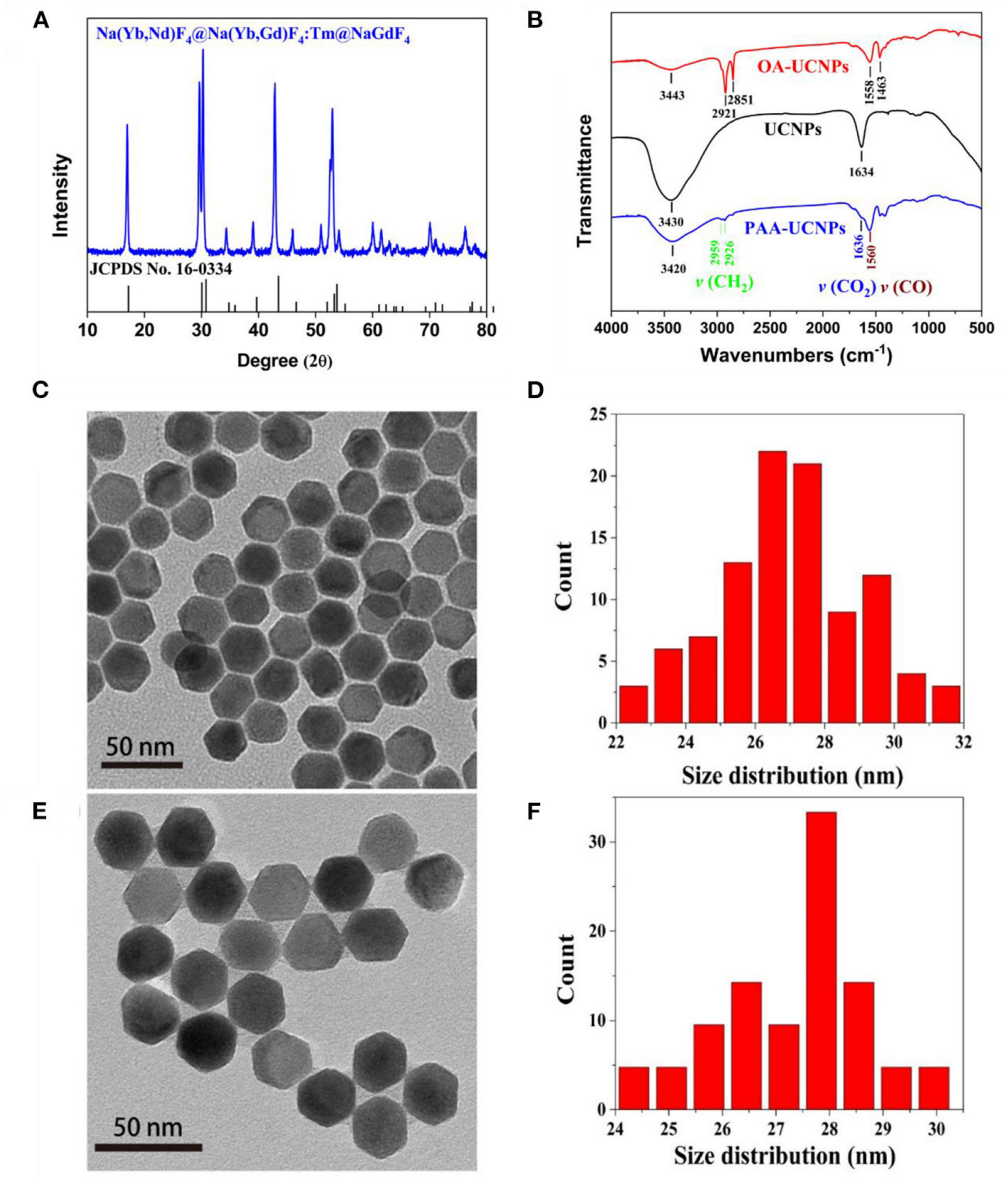
**(A)** XRD pattern of OA-capped NaYb_0.5_Nd_0.5_F_4_@NaYb_0.5_Gd_0.49_Tm_0.01_F_4_@NaGdF_4_ UCNPs in comparison with standard JCPDS No. 16-0334, **(B)** FT-IR spectra of the OA-capped, un-capped, and PAA-capped NaYb_0.5_Nd_0.5_F_4_@NaYb_0.5_Gd_0.49_Tm_0.01_F_4_@NaGdF_4_ UCNPs, and **(C,E)** TEM images; and **(D,F)** size distribution of the as-prepared OA-capped and PAA-capped NaYb_0.5_Nd_0.5_F_4_@ NaYb_0.5_Gd_0.49_Tm_0.01_F_4_@NaGdF_4_ UCNPs, respectively.

The structure and morphology characteristics of the as-prepared NaYb_0.5_Nd_0.5_F_4_@NaYb_0.5_Gd_0.49_Tm_0.01_F_4_@NaGdF_4_ UCNPs were validated by XRD, FT-IR and TEM techniques. The XRD pattern of the as-prepared OA-UCNPs, as shown in Figure 1A, matches well with the standard JCPDS No. 16-0334, which corresponds to the pure hexagonal structure of NaYF_4_. FT-IR technique validates the successful surface functionalization (i.e., the replacement of the OA with PAA) of the UCNPs. The FT-IR spectra of the OA-capped, un-capped, and PAA-capped UCNPs measured in the range of 500–4,000 cm^−1^ are presented in Figure 1B. The capping of the PAA molecules onto the surface of the UCNPs not only makes them water-dispersible but also provides multiple binding sites for detection of metal ions, specially Cu^2+^ ion. The FT-IR spectrum of the as-prepared OA-capped UCNPs exhibits five prominent peaks at 1,463, 1,558, 2,851, 2,921, and 3,443 cm^−1^. The presence of the doublets at (1,463, 1,558 cm^−1^) and (2,851 and 2,921 cm^−1^) attributes to the [symmetric (ν_s_) and asymmetric (ν_as_)] stretching vibrations of the carboxylic group (–COO) and methylene group (–CH_2_), respectively, which indicates the presence of OA ligand on the surface of the UCNPs (Chen et al., 2008; Dai et al., 2016; Wang et al., 2019). Also, a broad singlet observed at 3,443 cm^−1^ originates from the stretching vibration of the hydroxyl (–OH) group (Sarkar et al., 2015). For bare UCNPs (without OA on the surface), these doublets are disappeared from the FT-IR spectrum (black line in Figure 1B) of the uncapped UCNPs, indicating the successful removal of OA ligand from UCNPs surface after weak acidic treatment. Post OA removal, the UCNPs are further functionalized by the PAA and the FT-IR spectrum of the PAA-capped UCNPs is presented in Figure 1B (blue line), which clearly shows the characteristics doublet at 1,560 and 1,636 cm^−1^ assigned to asymmetric stretching vibrations of CO (ν_CO_), and CO_2_ (ν_C_O__2__) groups of PAA ligand, respectively (Liu et al., 2013; Feng et al., 2015; Kong et al., 2017). Another doublet at the higher wavenumber region, i.e., 2,926 and 2,959 cm^−1^ arises due to the symmetric and asymmetric stretching vibrations of CH_2_ (ν_C_H__2__), respectively. The intensity of the doublet caused by the CH_2_ (ν_C_H__2__) group decreases significantly in comparison to the OA capped UCNPs. Thus, we conclude that the capping of the PAA group onto the surface of the UCNPs is successful. This is consistent with the TGA results. The TGA plots of PAA-UCNPs and un-capped UCNPs are shown in Supplementary Figure 1 (Supporting Information). The TGA curves clearly show that the decomposition temperature for PAA-UCNPs at ~400 °C is higher than that of the pure PAA molecules at ~200 °C (Sarkar et al., 2015), indicating the strong affinity of PAA molecules to the UCNPs. TEM images of both OA-UCNPs and PAA-UCNPs confirm the formation of hexagonal morphology (see Figures 1C,E). Irrespective of the capping agent used, the particle size (estimated 27 ± 2.2 nm) and the morphology of the UCNPs are hardly affected, as evident from Figures 1C–F. The replacement of the OA by PAA onto the surface of UCNPs significantly improves the water dispersibility without any particle aggregation in PAA-UCNPs (Figure 1E). Supplementary Figure 2 (Supporting Information) shows the TEM images and size distribution of the as-prepared core of NaYb_0.5_Nd_0.5_F_4_ and core-shell structure with the composition of NaYb_0.5_Nd_0.5_F_4_@NaYb_0.5_Gd_0.49_Tm_0.01_F_4_ nanoparticles, respectively. The core and core-shell particles exhibit the particle size of 18 ± 0.5 and 25 ± 2.1 nm, respectively. Thus, the thickness is ~7 nm and ~2 nm for the inner shell and outmost shell layer, respectively.

### UV-Vis Absorption Spectra

**Figure 2:**
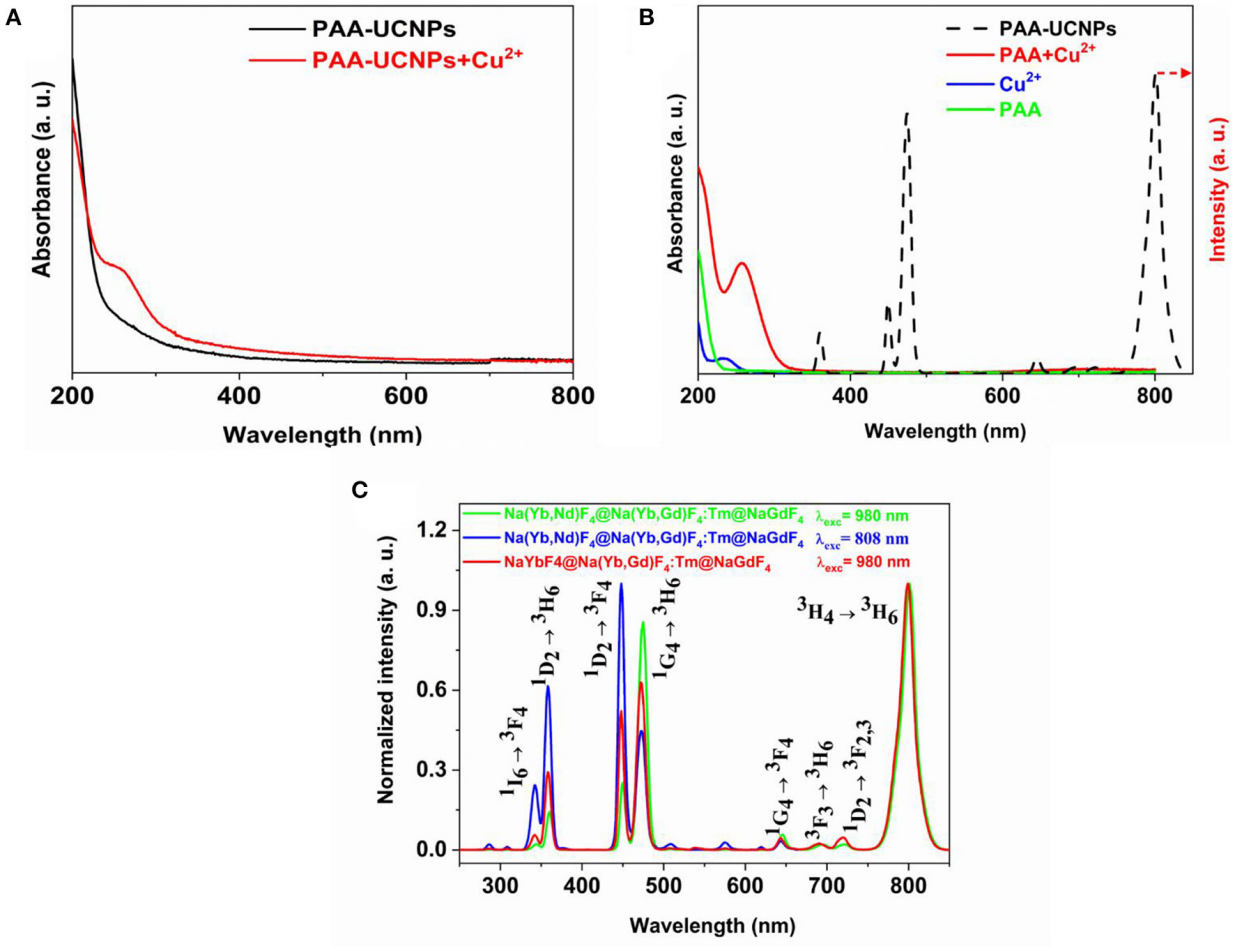
**(A)** UV-vis spectra of PAA capped NaYb_0.5_Nd_0.5_F_4_@NaYb_0.5_Gd_0.49_Tm_0.01_F_4_@NaGdF_4_ UCNPs and PAA capped UCNPs chelating with Cu^2+^; **(B)** UV-vis spectra of PAA, Cu^2+^, and PAA chelating with Cu^2+^, in comparison with upconversion emission spectra of PAA-capped NaYb_0.5_Nd_0.5_F_4_@ NaYb_0.5_Gd_0.49_Tm_0.01_F_4_@NaGdF_4_ UCNPs under 980 nm excitation; and **(C)** Normalized upconversion emission spectra of PAA capped NaYb_0.5_Nd_0.5_F_4_@NaYb_0.5_Gd_0.49_Tm_0.01_F_4_@NaGdF_4_ UCNPs under 980 and 808 nm laser excitation, in comparison to PAA capped NaYbF_4_@NaYb_0.5_Gd_0.49_Tm_0.01_F_4_@NaGdF_4_ UCNPs under 980 nm laser excitation.

The UV-Vis measurements were carried out to understand the sensing mechanism and the possibility of the interaction between the Cu^2+^ and PAA-UCNPs. The UV-Vis spectrum (Figure 2A) of the PAA-UCNPs does not show any absorption. In contrast, a noticeable absorption peak around 258 nm upon the addition of Cu^2+^ into PAA-UCNPs dispersion is due to the formation of the copper carboxylate complex based on PAA-UCNPs and Cu^2+^ interaction, in line with the reported literature (Schuetz and Caruso, 2003; Iatridi et al., 2008; Ma et al., 2008). This confirms the chelation between the Cu^2+^ and the carboxyl group of PAA on the surface of UCNPs. When mixing Cu^2+^ in PAA-UCNPs solution, the emission intensity of the UCNPs is expected to decline due to the formation of copper carboxylate complex. This could be explained either by FRET, inner-filter effect or an effect of the heavy metal ion. In order to rule out the FRET and inner-filter effect, the comparison between the UV-Vis spectra of the PAA, Cu^2+^, PAA+Cu^2+^, and upconversion emission spectrum of PAA-UCNPs has been presented in Figure 2B. Cu^2+^ ions exhibit a strong absorption band peaking at 234 nm. The interaction between the Cu^2+^ with PAA resulted in a strong, broadband at 260 nm along with a very weak broad band in the 600–800 nm region, which ascribe to –COO^−^-Cu^2+^ charge transfer (Ma et al., 2008; Sarkar et al., 2015), and the complexation of PAA with Cu^2+^ (Iatridi et al., 2008), respectively, with the latter band overlapping with the 645, 695, 723, 801 nm emission of the UCNPs. However, the intensity of overlapped peaks is very weak, in addition, the prominent emission peaks of UCNPs (i.e., at 344, 360, and 450, and 474 nm), are not overlapped. Hence, the FRET and inner-filter effect can be ruled out as a preferred fluorescence quenching mechanism (Wang et al., 2017). In these circumstances, we believed that the effect of heavy metal ion is the main reason for the fluorescence quenching of the UCNPs (Saleh et al., 2011; Liang et al., 2016).

### Upconversion Emission Spectra

Figure 2C shows the upconversion emission spectra of the PAA-capped Na(Yb,Nd)F_4_@Na(Yb,Gd)F_4_:Tm@NaGdF_4_ (excited at 980 and 808 nm, respectively), and NaYbF_4_@ Na(Yb,Gd)F_4_:Tm@NaGdF_4_ UCNPs (excited at 980 nm) UCNPs. The emission spectra displayed several sharp characteristics peaks of Tm^3+^ at 344, 360, 450, 474, 645, 695, 723, and 801 nm, which can be attributed to the ^1^I_6_ → ^3^F_4_, ^1^D_2_ → ^3^H_6_, ^1^D_2_ → ^3^F_4_, ^1^G_4_ → ^3^H_6_, ^1^G_4_ → ^3^F_4_, ^3^F_3_ → ^3^H_6_ (Wang et al., 2010; Liu et al., 2017; Zhan et al., 2017), ^1^D_2_ → ^3^F_2, 3_ (Zhao et al., 2013; Zhan et al., 2017) and ^3^H_4_ → ^3^H_6_ transitions of Tm^3+^, respectively (Kenyon, 2002; Wang et al., 2010, 2011; Zhou et al., 2013). All the emission spectra shown in Figure 2C are identical, except for their emission intensity. The appearance of UV/blue upconversion emissions indicates that the core-shell structure has successfully suppressed the deleterious cross-relaxation processes (Wen et al., 2013). Among the Tm^3+^ transitions, ^3^H_4_ → ^3^H_6_ (801 nm) transition shows significant higher emission intensity than the ^1^G_4_ → ^3^H_6_ (474 nm). The higher emission intensity of the ^3^H_4_ → ^3^H_6_ transition originates due to the depletion of ^3^H_4_ level by surface quenching, which will supress the population of ^1^G_4_ by subsequent excited state absorption or energy transfer (ET) (Wang et al., 2010). We have investigated the pump power-dependent upconversion luminescence with 980 nm excitation to figure out the actual number of photons involved in the upconversion process. Supplementary Figure 3 displays the plot of log(Intensity) vs. log(Laser power) (Supporting Information). The slope of the plot for 344 nm emission was 4.15, showing that the upconversion is five photon process. Under the 808 nm excitation, in comparison to the 980 nm excited Na(Yb,Nd)F_4_@Na(Yb,Gd)F_4_:Tm@NaGdF_4_, the emission intensity of the peaks below the 460 nm (UV and blue emissions) has increased with decrease in the 474 nm (^1^G_4_ → ^3^H_6_) emission peak, which agrees with the result as reported by Wen et al. (2013).

### Detection of Cu^2+^

**Figure 3:**
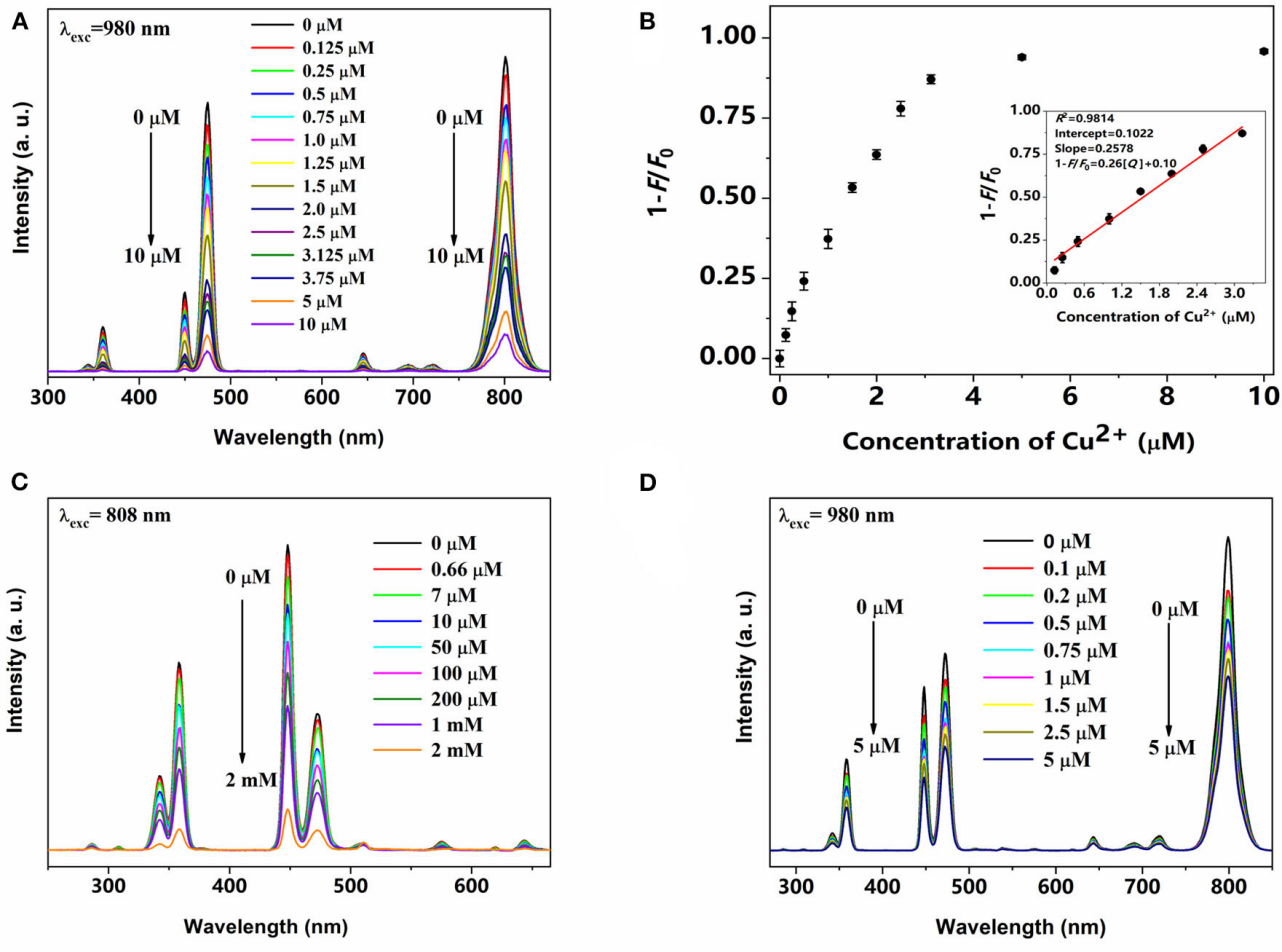
Upconversion emission spectra of PAA-NaYb_0.5_Nd_0.5_F_4_@NaYb_0.5_Gd_0.49_Tm_0.01_F_4_@NaGdF_4_ UCNPs with various concentration of Cu^2+^ under **(A)** 980 nm and **(C)** 808 nm laser excitation, respectively; **(B)** The plot of emission quenching efficiency (1-*F*/*F*_0_) vs. the Cu^2+^ concentration in (A); **(D)** Upconversion emission spectra of PAA-NaYbF_4_@NaYb_0.5_Gd_0.49_Tm_0.01_F_4_@NaGdF_4_ UCNPs with various concentration of Cu^2+^ under 980 nm laser excitation.

The emission spectra of PAA-NaYb_0.5_Nd_0.5_F_4_@NaYb_0.5_Gd_0.49_Tm_0.01_F_4_@NaGdF_4_ UCNPs under 980 nm excitation with different Cu^2+^ were measured and presented in Figure 3A. With the increase in the Cu^2+^ ions concentration, the emission intensity of the PAA-UCNPs decreased monotonically. To comprehend the effect of the Cu^2+^ on the luminescence quenching, a plot of emission quenching efficiency, 1-*F*/*F*_0_, where *F*_0_ and *F* denote the emission intensity of UCNPs without and with Cu^2+^ ions vs. the concentration of Cu^2+^ ions, respectively, is displayed in Figure 3B. The Cu^2+^ concentration and 1-*F*/*F*_0_ of PAA-UCNPs presents a linear relationship with *R*^2^ = 0.9814 within 0.125–3.125 μM range (Figure 3B), indicating the dynamic nature of the photoluminescence quenching (Sarkar et al., 2014). The fluorescence quenching efficiency reaches a plateau at higher concentration of Cu^2+^ after 4 μM, indicating the saturation of the chelating sites after Cu^2+^ binding (Wang et al., 2017). The LOD is calculated using the formula 3δ/*S* where δ denotes standard deviation and *S* is the slope. The LOD of Cu^2+^ ions using current PAA-UCNPs based sensor is as low as 0.1 μM, which is superior to that of existing upconversion Cu^2+^ detection methods (Sarkar et al., 2014; Huang et al., 2017). Also, the LOD of the current PAA-UCNPs platform is comparable with the values reported in the literature (Wang et al., 2014). The meso-tetra(4-sulfonatophenyl)porphine dihydrochlorid (TSPP) on SiO_2_-encapsulated NaYF_4_:Yb,Er,Gd UCNPs based platform for the detection of Cu^2+^ ions with a LOD of 2.16 μM was reported by the Huang et al. (2017). These results indicate that the current PAA-UCNPs based platform exhibits superior sensitivity for Cu^2+^ detection. The PAA-NaYb_0.5_Nd_0.5_F_4_@NaYb_0.5_Gd_0.49_Tm_0.01_F_4_@NaGdF_4_ UCNPs based platform has also shown quite high sensitivity even at 808 nm laser excitation within 0.66–2,000 μM range, indicating that the Cu^2+^ ions can also be effectively detected by using 808 nm laser, as shown in Figure 3C. In addition, the Cu^2+^ can also be detected by using the PAA-UCNPs having 100% Yb^3+^ in the core (NaYbF_4_@NaYb_0.5_Gd_0.49_Tm_0.01_F_4_@NaGdF_4_ UCNPs) under 980 nm excitation within 0.1–5 μM range, as can be seen in Figure 3D. The obtained results i.e., different probes, LOD, and linear range monitored at specific excitation wavelength in this study are compared with that of the reported literature for the Cu^2+^ detection using different nanoplatforms (probes), see Supplementary Table 1. To the best of our knowledge, the exact mechanism underlying the cause of quenching is still very much unclear because of highly complex nature of the upconversion luminescence. However, we can speculate about the quenching mechanism based on the observations reported in previous reports, i.e., dynamic and static quenching (Saleh et al., 2011). At low metal ion concentrations, the collisions between heavy metal ions and the UCNPs cause the dynamic quenching and form a complex between excited-state UCNPs and ground-state heavy metal ion during the excited-state lifetime of UCNPs. The excited state complex then loses its energy non-radiatively and falls to the ground state. On the other hand, the static quenching can also occurs at higher concentrations. In static quenching, the complex forms between the UCNPs and the heavy metal ions at ground state. Such a complex is very different from the excited-state complex and is temporally stable.

### Selectivity

**Figure 4:**
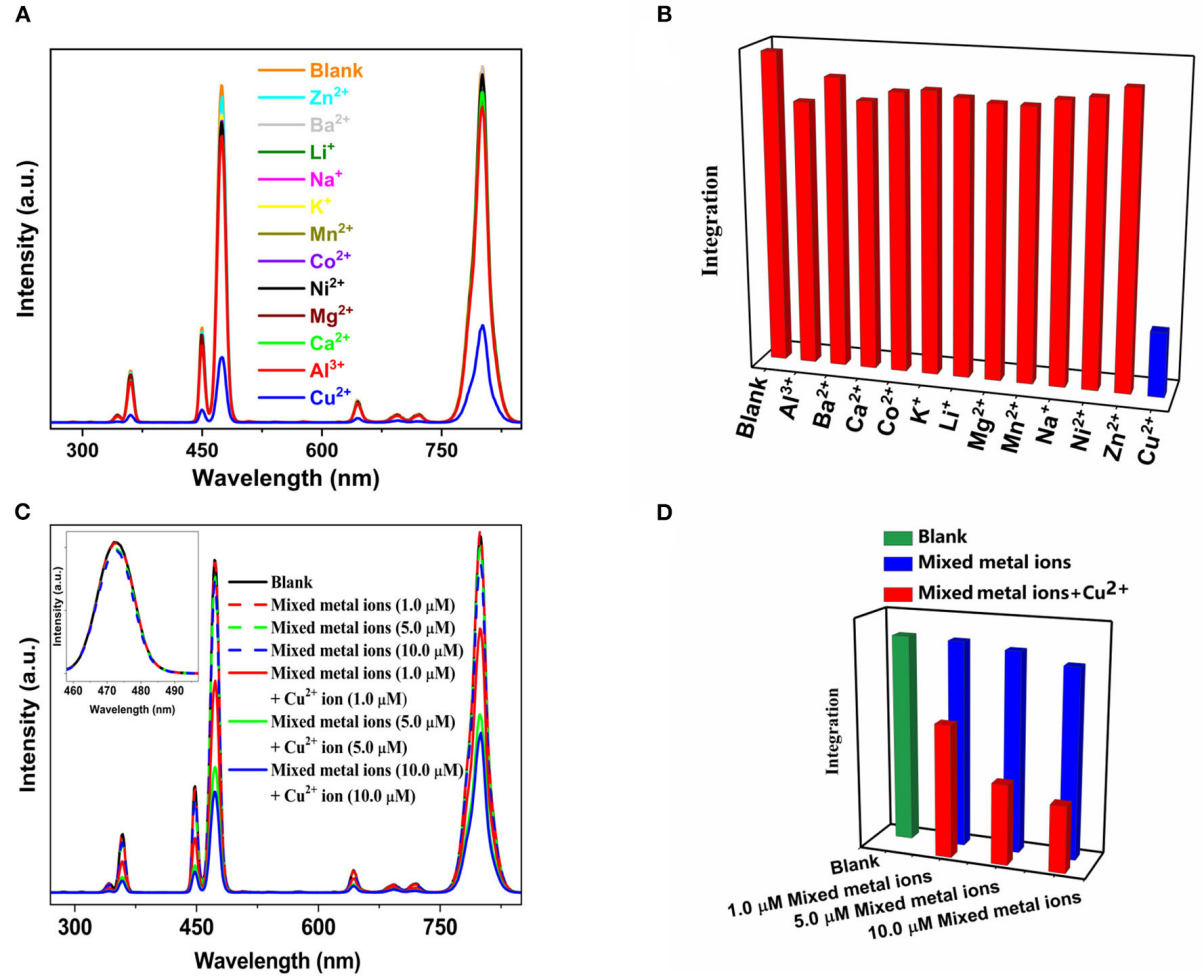
**(A)** The effect of various equimolar metal ions i.e., Al^3+^, Ba^2+^, Ca^2+^, Co^2+^, K^+^, Li^+^, Mg^2+^, Mn^2+^, Na^+^, Ni^2+^, Zn^2+^, and Cu^2+^ (2 μM) on the upconversion emission intensity of PAA-NaYb_0.5_Nd_0.5_F_4_@NaYb_0.5_Gd_0.49_Tm_0.01_F_4_@NaGdF_4_ UCNPs under 980 nm laser excitation; **(B)** graphical representation of **(A)**; **(C)** comparison of upconversion emission intensity of PAA-NaYb_0.5_Nd_0.5_F_4_@NaYb_0.5_Gd_0.49_Tm_0.01_F_4_@NaGdF_4_ UCNPs upon addition of mixed metals ions with and without Cu^2+^ ions under 980 nm laser excitation and **(D)** graphical representation of **(C)**.

The selectivity of the PAA-NaYb_0.5_Nd_0.5_F_4_@NaYb_0.5_Gd_0.49_Tm_0.01_F_4_@NaGdF_4_ UCNPs under 980 nm excitation toward Cu^2+^ ions was further investigated by testing the PAA-UCNPs response toward a wide range of metal ions, particularly, equimolar Al^3+^, Ba^2+^, Ca^2+^, Co^2+^, K^+^, Li^+^, Mg^2+^, Mn^2+^, Na^+^, Ni^2+^, and Zn^2+^. Figure 4A shows the response of the PAA-UCNPs toward these metal ions, shown by individual red column for each metal ion, in comparison to the PAA-UCNPs (blank) and Cu^2+^ ions response in Figure 4B. It is evident that the emission intensity of blank sample after the addition of various metal ions has not been significantly altered, except for the Cu^2+^, indicating that the current PAA-UCNPs based sensing platform is most sensitive toward the Cu^2+^ ions. Furthermore, to confirm the competitive selectivity of the Cu^2+^ ion by PAA-UCNPs, the upconversion emission spectra of PAA-UCNPs in presence of mixed metal ions i.e., Al^3+^, Ba^2+^, Ca^2+^, Co^2+^, K^+^, Li^+^, Mg^2+^, Mn^2+^, Na^+^, Ni^2+^, and Zn^2+^ at different concentrations (1, 5, 10 μM) were measured and presented in Figure 4C. It can be seen that the mixture of the metal ions in absence of the Cu^2+^ do not show substantial difference in the emission intensity, however, as the Cu^2+^ ions were added to the solution containing other metal ions, the emission intensity was significantly decreased monotonically with increase in the Cu^2+^ concentration, see Figure 4D. The decrease in the upconversion emission intensity can be ascribed to the chelation of Cu^2+^ and PAA-UCNPs that leads to the efficient fluorescence quenching of PAA-UCNPs. These results demonstrate that the PAA-UCNPs based platform can serve as an upconversion detector for the detection of the Cu^2+^ with high sensitivity and selectivity. The preferred selectivity of the Cu^2+^ ions can be explained by considering the binding constants of the multi-valent ions to the PAA. Many researchers have measured the binding constants of the different multivalent metal ions and reported in the literature (Mandel and Leyte, 1964; Porasso et al., 1999; Giesecke et al., 2016). According to these reports, the selectivity of the metal ion not only depends on the pH of the solution but also on the ionic radii and their concentration. The reported trend for the different multivalence metal ions based on their binding constants is as follows, Li^+^ < Na^+^ < K^+^≈Ba^2+^ < Ca^2+^≈Mg^2+^ < Al^3+^ < Co^2+^ < Ni^2+^ < Zn^2+^ < Mn^2+^ < Cu^2+^. Since the Cu^2+^ shows the highest binding constant values, it forms stronger coppercarboxylate complex and thus greatly quenches the luminescence.

### Reversibility

**Figure 5:**
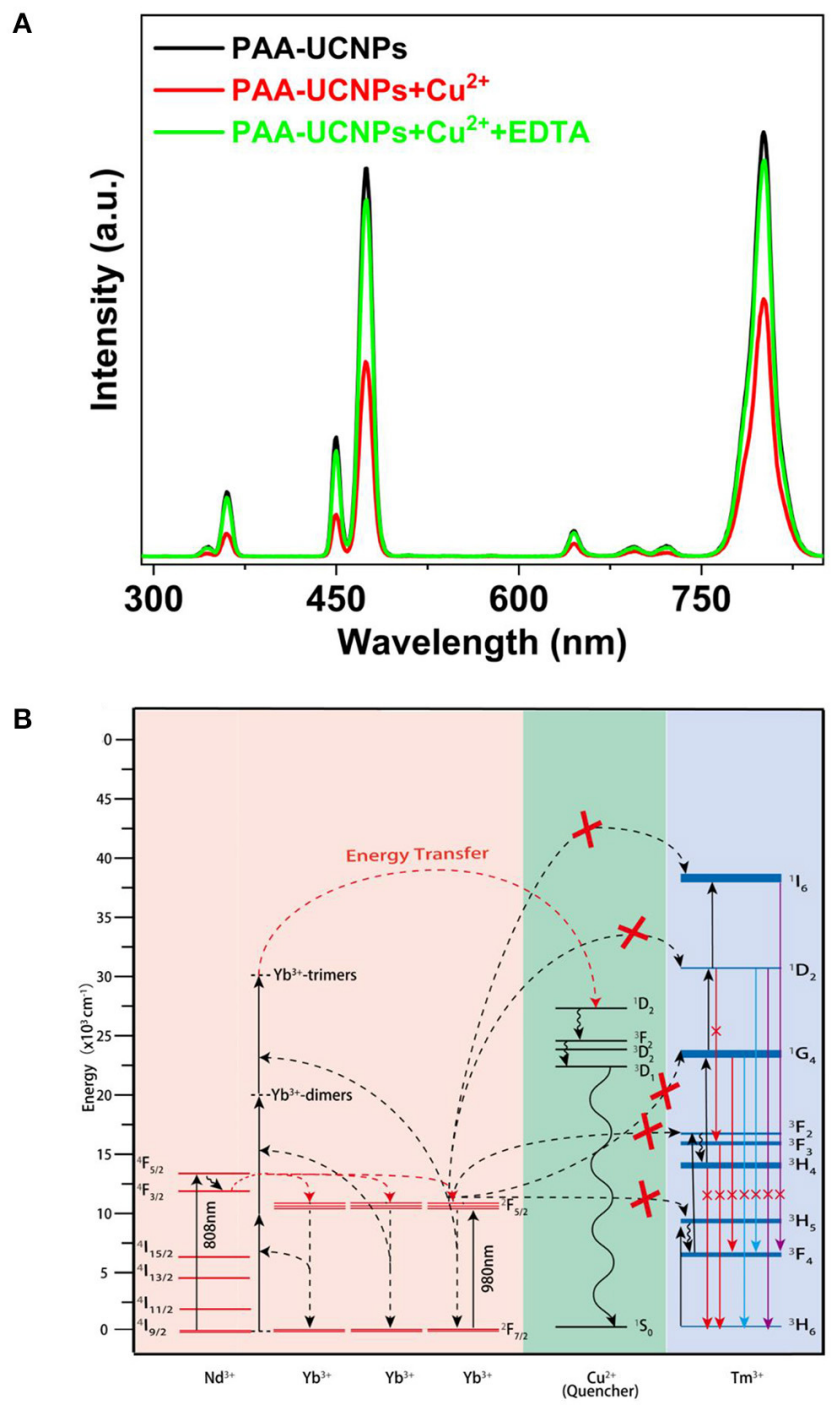
**(A)** Photoluminescence spectra of PAA-NaYb_0.5_Nd_0.5_F_4_@NaYb_0.5_Gd_0.49_Tm_0.01_F_4_@NaGdF_4_ UCNPs without Cu^2+^, with Cu^2+^ (2.0 μM), and further addition of EDTA (2.0 μM) under 980 nm laser excitation; **(B)** The proposed energy transfer mechanism between NaYb_0.5_Nd_0.5_F_4_@NaYb_0.5_Gd_0.49_Tm_0.01_F_4_@NaGdF_4_UCNPs and copper ions.

We also checked the reversibility of the Cu^2+^ ions detection using PAA-NaYb_0.5_Nd_0.5_F_4_@NaYb_0.5_Gd_0.49_Tm_0.01_F_4_@NaGdF_4_ UCNPs. After addition of the Cu^2+^ to the PAA-UCNPs, post copper carboxylate complex formation, EDTA was added gradually for this purpose. Upon 980 nm excitation, the emission spectra of the PAA-UCNPs before (black line) and after (red line) the Cu^2+^ addition with obvious emission intensity difference, are shown in Figure 5A. Upon the gradual addition of EDTA to the copper carboxylate complex, the emission intensity of the PAA-UCNPs is gradually recovered (green line) up to 90%, suggesting the PAA-UCNPs based platform for the Cu^2+^ detection is reversible. It is also noted that the PAA-UCNPs based platform responds very quickly within a minute for both the Cu^2+^ ions to quench the emission and the EDTA solution to recover the quenched emission. We believe that the strong affinity of the Cu^2+^ toward EDTA is responsible for the dissociation of the copper carboxylate complex thereby recovering the emission intensity of the PAA-UCNPs.

### Proposed Energy Transfer Mechanism

Figure 5B demonstrates the energy level diagram depicting plausible mechanism of upconversion emission quenching caused by the addition of Cu^2+^ to PAA-UCNPs. Generally, upon 980 nm excitation, the Yb^3+^ ions are excited from their ^2^F_7/2_ ground state to the ^2^F_5/2_ excited state, subsequently, the cooperative ET from Yb^3+^-trimer to the different level of the Tm^3+^ produces the visible emission (Jean-Claude and Claude, 2002; Saleh et al., 2011; Wen et al., 2013; Qin et al., 2014). However, in presence of the Cu^2+^ ions, the cooperative ET from Yb^3+^-trimer to Tm^3+^ is hampered by the quenching processes caused by Cu^2+^, which leads to the upconversion emission quenching of the PAA-UCNPs (Saleh et al., 2011). We strongly believe that the significant quenching of the upconversion emission of PAA-UCNPs upon Cu^2+^ addition, is caused by the disturbance of the Yb^3+^ → Tm^3+^ ET by the heavy metal ion. Upon adding EDTA, the Cu^2+^ ions leave the copper carboxylate complex to bind with the EDTA because of their higher affinity toward EDTA than the PAA and thus the upconversion emission gets recovered.

## Conclusions

We have developed a simple water-dispersible PAA-UCNPs based nanoplatform for the detection of Cu^2+^ ions. With increase in the Cu^2+^ concentration, the upconversion emission intensity of the PAA-UCNPs is quenched monotonically within the 0.125–3.125 μM range due to the ET between PAA-UCNPs and Cu^2+^. The LOD for the Cu^2+^ is calculated to be 0.1 μM. It has been found that the PAA-UCNPs based nanoplatform can detect Cu^2+^ with higher sensitivity and selectivity even from the mixture of the various metal ions under 980 nm laser excitation. We believe that the PAA-UCNPs based nanoplatform under NIR excitation can be used in a wide range of practical bio-applications due to their superior characteristics such as no autofluorescence from bio-samples and interference from scattered excitation source, and high penetration depth. This makes PAA-UCNPs based nanoplatform a promising candidate for the biological applications in copper ion-related diseases i.e., in detection and diagnosis.

## Data Availability Statement

The original contributions presented in the study are included in the article/Supplementary Material, further inquiries can be directed to the corresponding author/s.

## Author Contributions

HW conceived the study. SS, HW, and XC designed the experiments. HW and DAH wrote the manuscript. SS, ZM, and GT carried out the material synthesis, characterization, and measurements. SS, HW, XC, and DAH analyzed the data. All authors contributed to the article and approved the submitted version.

## Conflict of Interest

The authors declare that the research was conducted in the absence of any commercial or financial relationships that could be construed as a potential conflict of interest.
