# Supplementary material for: PAA Modified Upconversion Nanoparticles for Highly Selective and Sensitive Detection of Cu2+ Ions
Source: Front Chem. 2021 Jan 8;8:619764. doi: 10.3389/fchem.2020.619764 (PMC7821086; doi:10.3389/fchem.2020.619764)
Supplement: Supplementary file 1 [file Data_Sheet_1.PDF]

PAA modified upconversion nanoparticles for highly selective and sensitive detection of Cu^{2+} ions

Shaoshan Su, Zhurong Mo, Guizhen Tan, Hongli Wen*, Xiang Chen*, D. A. Hakeem*

Key Laboratory of Clean Chemistry Technology of Guangdong Regular Higher Education Institutions, School of Chemical Engineering and Light Industry, Guangdong University of Technology, Guangzhou 510006, P.R. China

*Corresponding author:

E-mail addresses: hongliwen@gdut.edu.cn (H. Wen); chenxiang@gdut.edu.cn (X. Chen); abdulhakeem.desh@gdut.edu.cn (D. A. Hakeem)

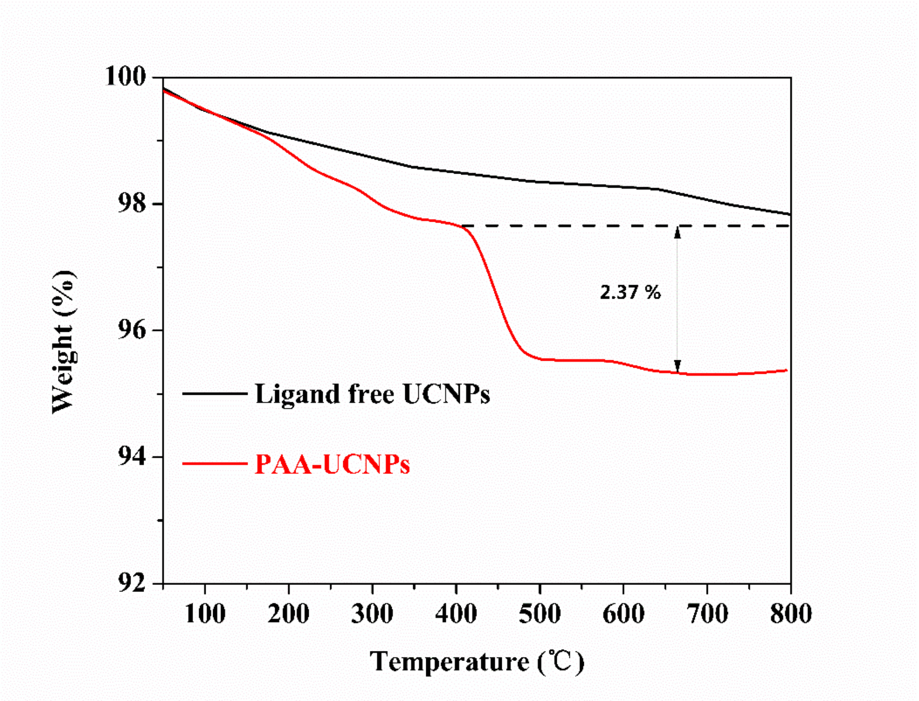

Fig. S1 TGA curves of ligand free $NaYb_{0.5}Nd_{0.5}F_4@NaYb_{0.5}Gd_{0.49}Tm_{0.01}F_4@NaGdF_4$ UCNPs and PAA coated $NaYb_{0.5}Nd_{0.5}F_4@NaYb_{0.5}Gd_{0.49}Tm_{0.01}F_4@NaGdF_4$ UCNPs, respectively.

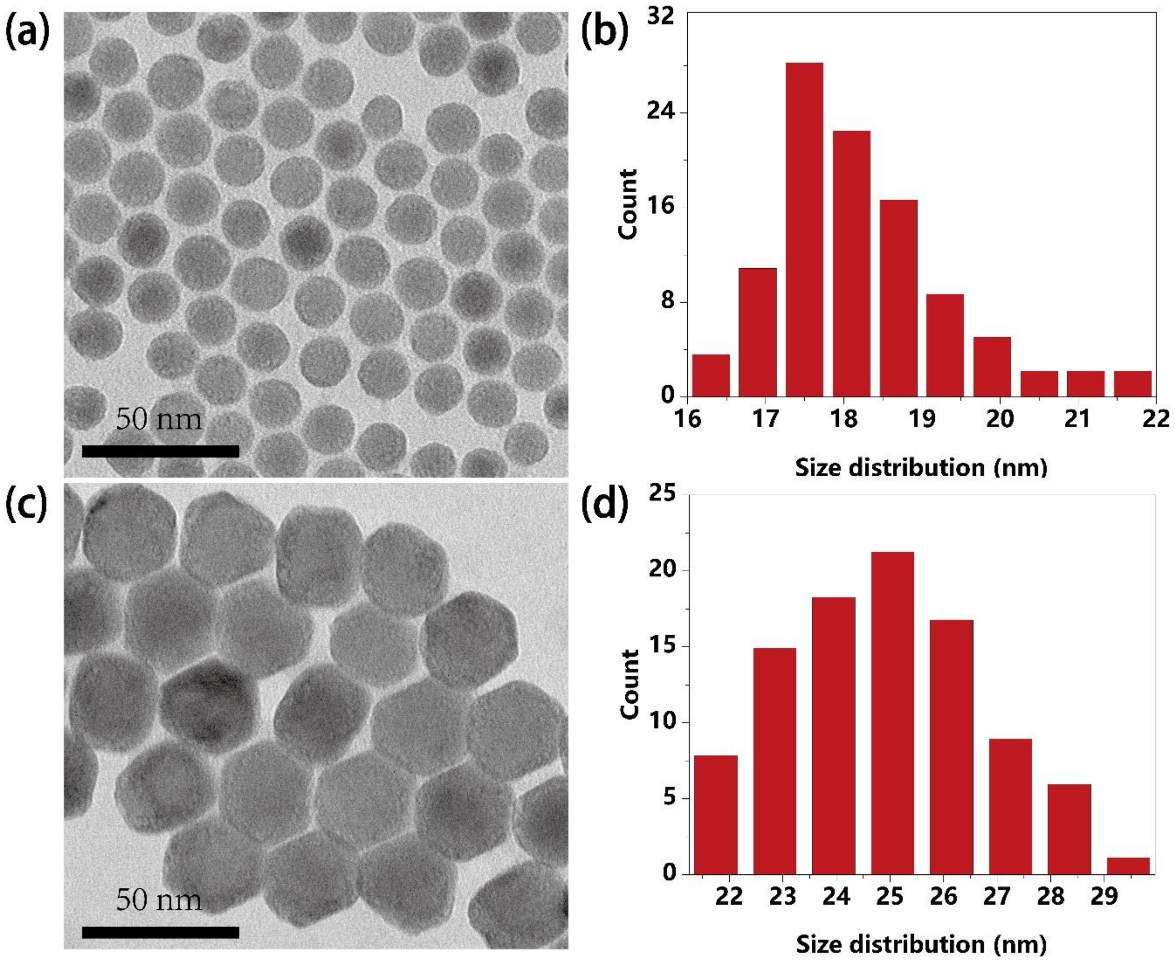

Fig. S2 TEM images (a and c) and size distribution (b and d) of the as-prepared core of $NaYb_{0.5}Nd_{0.5}F_4$ and core-shell structure with composition of $NaYb_{0.5}Nd_{0.5}F_4$@$NaYb_{0.5}Gd_{0.49}Tm_{0.01}F_4$ nanoparticles, respectively.

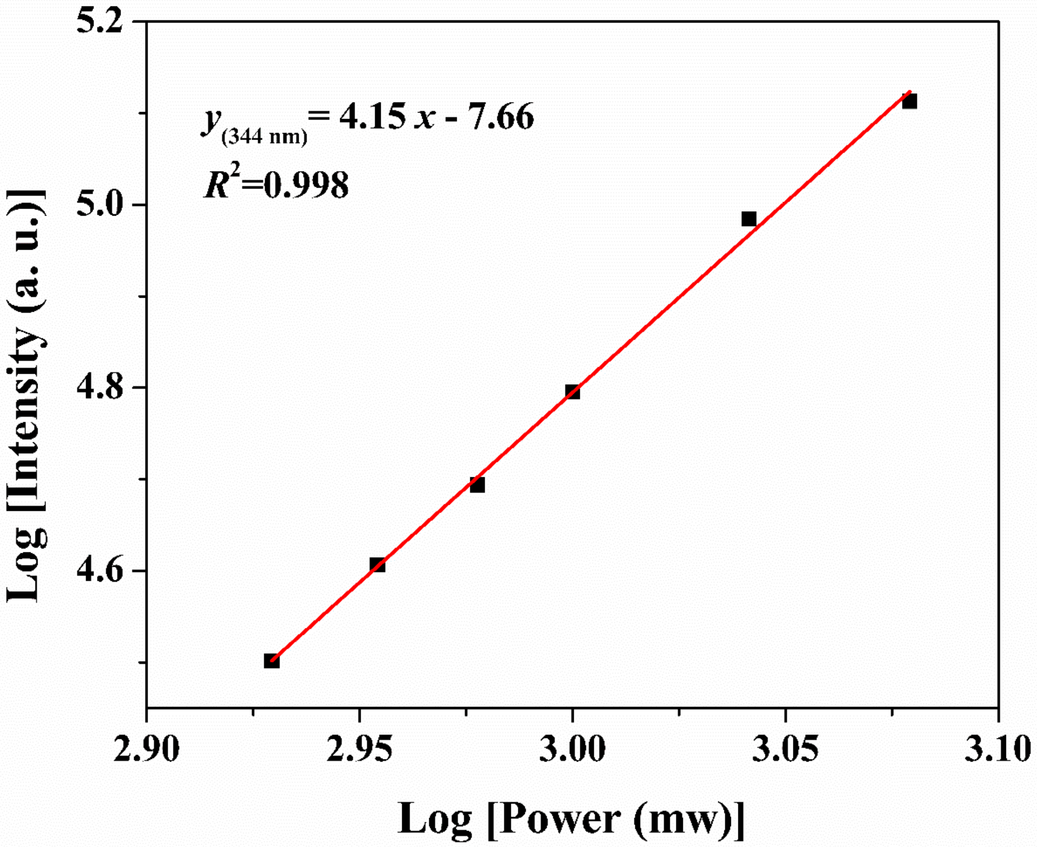

Fig. S3 Double-logarithmic plots of the pump-power dependent upconversion emission intensity from Tm^{3+} emission at 344 nm recorded under 980 nm excitation.

Table S1. Comparison of the PAA-UCNPs nanoplatform with the other reported platforms for the Cu^{2+} detection.

**Probes**	**Analytes/excitation wavelength**	**LOD**	**Linear range**	**reference**
PAA-$NaYb_{0.5}Nd_{0.5}F_4$@$NaYb_{0.5}Gd_{0.49}Tm_{0.01}F_4$@$NaGdF_4$	Cu^{2+}/ 980 nm	0.1 μM	0.125–3.125 μM	This work
TPEA-Carbon quantum dots	Cu^{2+}/ 405 nm	10 nM	10^{-6} –10^{-4} M	1
Rhodamine B-SiO_2 coated $NaYF_4$:Yb,Er@$NaYF_4$	Cu^{2+}/ 520 nm	0.82 μM	-	2
Carboxyl-modified CdTe QDs	Cu^{2+}/ 340 nm	0.36 nM	0 to 100 nM	3
PEI-capped $NaGdF_4$:Yb/Tm	Cu^{2+}/ 980 nm	57.8 nM	0.1-2 μM	4
TSPP-$NaYF_4$:Yb,Er,Gd@SiO_2	Cu^{2+}/ 980 nm	2.16 μM	5 μM–0.16 mM	5
Rd-NH_2-β-$NaYF_4$:Yb^{3+}/Er^{3+}	Cu^{2+}/ 980 nm	-	2–14 μM	6
RB-hydrazide-$NaYF_4$:Yb^{3+}/Er^{3+}	Cu^{2+}/ 980 nm	10^{-6} M	10^{-5} to 10^{-6} M	7

*TPEA = ([N-(2- aminoethyl)-N,N,N’ -tris(pyridin-2-ylmethyl)
TSPP = meso-tetra(4-sulfonatophenyl)porphine dihydrochloride
Rd-NH_2 = 2-amino-3′,6′-bis(ethylamino)-2′,7′-dimethyl-3′,9a′-dihydrospiro[isoindoline-1,9′-xanthen]-3-one

References:

1. Q. Qu, A. Zhu, X. Shao, G. Shi and Y. Tian, Development of a carbon quantum dots-based fluorescent Cu^{2+} probe suitable for living cell imaging, Chem. Commun., 48(2012)5473-5475.
2. Y.X. Xu, H.F. Li, X.F. Meng, J.L. Liu, L.N. Sun, X.L. Fan, L.Y. Shi, Rhodamine-modified upconversion nanoprobe for distinguishing Cu^{2+} from Hg^{2+} and live cell imaging, New J. Chem., 40(2016)3543-3551.
3. Y.H. Wang, C. Zhang, X.C. Chen, B. Yang, L. Yang, C.L. Jiang, Z.P. Zhang, Ratiometric fluorescent paper sensor utilizing hybrid carbon dots–quantum dots for the visual determination of copper ions, Nanoscale, 8(2016)5977-5984.
4. F. Wang, C. Zhang, Q. Xue, H. Li, Y. Xian, Label-free upconversion nanoparticles-based fluorescent probes for sequential sensing of Cu^{2+}, pyrophosphate and alkaline phosphatase activity, Biosens. Bioelectron., 95(2017)21-26.
5. X. Huang, L. Wang, X. Zhang, X. Yin, N. Bin, F. Zhong, et al., Dye-assembled nanocomposites for rapid upconversion luminescence sensing of Cu^{2+}, Sens. Actuators B Chem., 248(2017)1-8.
6. X. Jiang, G. Meng, A rhodamine-based sensing probe excited by upconversion $NaYF_4$:Yb^{3+}/Er^{3+} nanoparticles: The realization of simple Cu(II) detection with high sensitivity and unique selectivity, J. Lumin., 135(2013)227-231.
7. J. Zhang, B. Li, L. Zhang, H. Jiang, An optical sensor for Cu(ii) detection with upconverting luminescent nanoparticles as an excitation source, Chem. Commun., 48(2012)4860-4862.
